# Tomato defences modulate not only insect performance but also their gut microbial composition

**DOI:** 10.1038/s41598-023-44938-2

**Published:** 2023-10-24

**Authors:** Andreea Bosorogan, Erick Cardenas-Poire, Eliana Gonzales-Vigil

**Affiliations:** 1 Department of Biological Sciences, University of Toronto Scarborough, Toronto, M1C 1A4 Canada; 2 Department of Cell and Systems Biology, University of Toronto, Toronto, M5S 3G5 Canada; 3 Microbiome Insights, Richmond, V6V 2G3 Canada

**Keywords:** Ecology, Plant sciences

## Abstract

Plants protect their tissues from insect herbivory with specialized structures and chemicals, such as cuticles, trichomes, and metabolites contained therein. Bacteria inside the insect gut are also exposed to plant defences and can potentially modify the outcome of plant–insect interactions. To disentangle this complex multi-organism system, we used tomato mutants impaired in the production of plant defences (*odorless-2* and *jasmonic acid–insensitive1*) and two cultivars (Ailsa Craig and Castlemart), exposed them to herbivory by the cabbage looper (*Trichoplusia ni* H.) and collected the insect frass for bacterial community analysis. While the epicuticular wax and terpene profiles were variable, the leaf fatty acid composition remained consistent among genotypes. Moreover, larval weight confirmed the negative association between plant defences and insect performance. The distinctive frass fatty acid profiles indicated that plant genotype also influences the lipid digestive metabolism of insects. Additionally, comparisons of leaf and insect-gut bacterial communities revealed a limited overlap in bacterial species between the two sample types. Insect bacterial community abundance and diversity were notably reduced in insects fed on the mutants, with Enterobacteriaceae being the predominant group, whereas putatively pathogenic taxa were found in wildtype genotypes. Altogether, these results indicate that plant defences can modulate insect-associated bacterial community composition.

## Introduction

Plants and insect herbivores are involved in an evolutionary arms race. Upon feeding, insect herbivores are subject to plant structural and chemical defences that delay their development and reproduction. To counteract the effects of plant defences, insects have developed mechanisms to avoid and/or detoxify plant defence metabolites^1–5^. This has led to an escalation of metabolic diversity in plants^6,7^. Notably, plant- and insect-associated bacteria also play roles in modulating the outcome of plant–insect interactions^8^. Examples show that insect-gut associated bacteria can suppress plant antiherbivore defences and degrade toxic plant specialized compounds; meanwhile, plant-associated bacteria can aid in plant resistance by slowing insect growth^4,9,10^. However, insect herbivores frequently host advantageous bacterial endosymbionts that play roles in facilitating their growth and enhancing their stress resistance^11^. Previous studies have shown that caterpillar gut bacterial communities can be influenced by the plant species the insect feeds on and environmental factors^8,12^. Yet, how different genotypes of the same plant with varying levels of defences impact the structure of bacterial communities is vastly unknown.

From the first contact with plants, insects are targeted by plant preformed specialized metabolites and structures. The aerial surface of all plants is covered by a protective epicuticular wax layer composed of several major classes of lipidic components such as alkanes, alcohols, free fatty acids, and triterpenoids. The chemical composition of this surface can alter the feeding and locomotion of insect herbivores^13–16^. For example, cabbage loopers, aphids (*Acyrthosiphon pisum* H.), and diamondback caterpillars (*Plutella xylostella* L.) display increased locomotion and lower falling frequencies in plants with reduced wax abundance^14^. Additionally, plant epidermal surfaces can be covered by hair-like structures (trichomes) that aid in resistance against insect herbivores. Non-glandular trichomes can hinder the movement of small insects and rupture their gut peritrophic matrix upon ingestion, whereas glandular trichomes produce chemical exudates that negatively impact insect growth and development^17–20^. Tomato leaves are abundant in glandular trichomes which accumulate terpenes, acylsugars, and anthocyanins^6,21,22^. These chemicals are toxic to insects and the rupture of glandular trichomes by caterpillars can induce the expression of defensive genes ^23^.

Once insect herbivores bypass constitutive defences, they are exposed to inducible defence mechanisms that are coordinated predominantly by the lipid-based phytohormone jasmonic acid (JA) and its derivatives. The cascade of responses is initiated by perception of damage-associated molecular patterns (DAMPs) released from insect oral secretions and damaged plant tissue, that in turn induce JA biosynthesis in plants^6,24^. JA is conjugated with isoleucine to form the bioactive jasmonoyl-L-isoleucine (JA-Ile), which binds to the *CORONATINE-INSENSITIVE1* (*COI1)-JASMONATE ZIM-domain* coreceptor, and induces transcriptional changes that lead to increased production of plant chemical and structural defences against herbivores^25,26^.

Considering the abundance of plant defensive strategies that herbivores are exposed to, it is crucial to understand whether and how insect gut bacterial communities are affected by their presence. Plant terpenes can disturb gut chemiosmosis and enhance the crossover of plant toxins from the gut to the hemolymph, altering the composition and abundance of insect-associated bacteria^27,28^. Sakuranetin, a JA-induced specialized metabolite from rice, was shown to limit the growth of beneficial endosymbionts in an Hemipteran insect^29^. However, most Lepidopteran gut bacterial microbiomes are relatively simple and consist of a few dominant bacterial taxa, predominantly belonging to the genera *Enterobacter*, *Pseudomonas*, and *Enterococcus*^30,31^. *Enterobacter* spp. can produce enzymes that help Lepidopteran species to detoxify plant phenolics, as well as provide essential amino acids that insects cannot synthesize^32^. Members of *Pseudomonas* can detoxify alkaloids, which are JA-regulated specialized metabolites, in the gut of two Lepidopteran species^33^. Gut bacteria from the genus *Bacillus, Enterococcus*, and *Staphylococcus* can help overcome chemical induced defenses with their protease activity^31^. Despite the wide distribution of microbes in the environment, interspecific differences in the gut bacterial composition between two Lepidopteran species consuming the same plant host have been seen. This implies that internal selective forces that influence the gut bacterial microbiome structure are at play^12^. While these findings highlight the importance and roles of insect gut bacteria, the extent to which plant defences affect the insect gut bacterial microbiome is still to be determined. Therefore, comprehensive approaches that examine the tripartite relationship by assessing the chemical composition of plant hosts, insect herbivore performance, and bacterial communities are needed.

Here, we use the interaction between tomato (*Solanum lycopersicum* L.) and the cabbage looper (*Trichopulsia ni* H.: Lepidoptera) to determine if bacterial communities associated with the insect digestive tract change in response to plant defences on the host plant. To manipulate the levels of plant defences, we used two tomato cultivars (Ailsa Craig and Castlemart) and two tomato mutants, *jasmonic acid-insensitive1* (*jai1*) and *odorless-2* (*od-2*), with reduced levels of plant defences^34,35^. The largest defect in plant defences is seen in the tomato *jai1*, which contains a knock-out mutation on the *COI1* gene. Hence, *jai1* mutants are highly susceptible to insect herbivores due to the inability to induce all jasmonate-related defences, including protease inhibitors, reduced trichomes and terpenes^35,36^. In contrast, the tomato *od-2* mutant has phenotypes associated with constitutive epidermal defences, including lower density and distorted type VI glandular trichomes, trace amounts of trichome-borne volatile compounds and flavonoids. Whereas the accumulation of acylsugars, glycoalkaloids, and protease inhibitors induced by JA are not affected. The defects make *od-2* susceptible to Coleopterans and Hemipterans^34,37^.These mutants provided two levels of defects in plant defences for testing the impact on the associated microbiome. We used a combination of metabolite profiling of lipid-derived compounds, insect bioassays and 16S rRNA gene sequencing to ask if microbial communities inside the gut change in response to plant defences. Our results highlight the significance of including the microbiome in plant–insect studies, recognizing that the microbiome is an active participant that responds to plant defences.

## Methods

### Plant growth conditions

Seeds of tomato (*S. lycopersicum*) cv. Castlemart (CT), cv. Ailsa Craig (AC), the mutants *od-2* and *jai1* (in the CT background) were kindly provided by Dr. Gregg Howe (Michigan State University, USA), as per the seed import guidelines provided by the Canadian Food Inspection Agency. Seeds were surface sterilized with chlorine gas (97% bleach and 3% HCl) and germinated under sterile conditions in the dark. The *jai1* homozygous seedlings were selected by application of 1 mM methyl-jasmonate (MeJA, PhytoTechnology Laboratories), as previously described^35^. The *jai1* homozygotes were identified based on their longer roots and the absence of purple hypocotyls, and further confirmed by PCR. Five days post germination, seedlings were transferred to autoclaved substrate (1:1 Pro-Mix PGX: HP MYCORRHIZAE) and grown in a growth chamber set at 26°C (12h with light) and 22°C (12h darkness). Four-week-old plants were moved into the greenhouse at the University of Toronto—Scarborough with a daily average temperature of 25.1°C and 32.5% humidity. This study complies with local and national guidelines. As *S. lycopersicum* is a commonly grown vegetable, no permission is required to collect or grow it.

### Insect rearing

*T. ni* eggs were obtained from the Insect Production Services (Great Lakes Forestry Centre, Sault Ste. Marie, ON, Canada) and hatched at room temperature^38^. Caterpillars were reared on five-week-old plants of four different genotypes (AC wildtype, CT wildtype, *jai1*, *od-2*), from neonate stage until they reached the last instar stage (approximately two weeks). Two butterfly cages were set up per genotype, each containing eight plants and thirty newly hatched caterpillars. Caterpillar weight was recorded at several time points throughout the experiment and insects were returned to the same cage after measurements.

### Foliar volatile terpene analysis

To better control the amount of insect damage, plants used for metabolite analysis were treated by caging one second- or third-instar insect to the fifth youngest leaf. The leaf immediately above (fourth youngest) was used for all metabolite analysis. From this leaf, different leaflets were used for terpene and cuticular wax analysis. Three to four replicates per genotype were used. Analysis of volatile terpenes was conducted as previously described with some modifications^39,40^. One leaflet was dipped in 1 mL of hexane containing 10 μg/mL of tetradecane (Alfa Aesar) as internal standard and gently shaken for 5 min at room temperature. The solvent was transferred to a 2mL-glass vial and leaflets were dried overnight in an oven before measuring dry weight. Samples were run on the Agilent 6890N Series GC-FID (gas chromatography-flame ionization detector) System (Agilent Technologies). Separation was achieved by injecting 1μl of hexane extract into an HP-1 column (30 m × 0.32 mm × 1.00 μm; Agilent) using the following temperature profile: 50 °C for 2 min; 5 °C/min to 230 °C; 45 °C/min to 300 °C with 5 min hold time. For peak identification, a representative sample was run on a GC–MS. Mono- and sesquiterpene compounds were identified by comparing their mass spectra to a volatile library^41^. Peaks areas were normalized to the internal standard and dry leaf weight.

### Cuticular wax profiling

For wax extraction, a second leaflet from the same leaf used for terpene analysis was collected. Leaflet areas were scanned prior to being stored at −80 °C. Cuticular wax extraction was performed as per previous protocols^42^. Leaflets were dipped in 10 mL of chloroform containing 1 μg/μL of the internal standard tetracosane (Agilent Technologies). Separation was achieved by injecting 1 μL into a GC-FID equipped with a HP-1 column (30 m × 0.32 mm × 1.00 µm; Agilent) using the following temperature profile: 50 °C for 2 min; 45 °C/min to 200 °C with 1 min hold time; 4 °C/min to 300 °C with 10 min hold time. Peaks were identified as described above and peak areas were normalized to the internal standard and leaflet surface area.

### Fatty acid methyl ester analysis

Fatty acid methyl esters (FAME) were extracted as per previous protocols^43^, with minor modifications. Briefly, frass and leaf samples were ground to powder with liquid nitrogen and 40 mg weighed in glass vials. Each frass and leaf sample had three technical replicates. To each vial, 2 mL of a 1.5 M H_2_SO_4_ solution containing 5 μg/mL nonadecanoic acid (internal standard; Agilent Technologies) was added, then heated to 85°C for 1.5 h with periodic mixing. After cooling, pentane and 0.9% NaCl (1:1) was added to separate methyl esters. The organic phase was analyzed by an Agilent 5977A Series GC-MSD (GC-mass spectrophotometer detector) fitted with a 30 m × 0.25 mm × 0.25 μm HP-5 column using the following temperature program: 8 °C/min from 100 to 250 °C with 10 min hold time. Compounds were identified by comparing their spectra against the NIST library, and peak areas were integrated using MassHunter Quantitative Analysis (Agilent Technologies). Areas were normalized to the internal standard and ground weight.

### Leaf- and insect-associated bacteria amplicon sequencing

Frass (insect feces) was used as a proxy for insect gut-associated bacteria. This choice was supported by previous studies on cabbage-fed *T. ni* that showed similar bacterial communities across different organs (i.e., the alimentary canal, Malpighian tubules, and mandibular glands)^44^. Similarly, in *Lymantria dispar* grown in the wild and the lab, small differences were found in bacterial abundance in frass and gut^45^. To avoid environmental contamination of the samples during frass collection, insects from each cage were transferred to individual sterile containers for 4–6 h. The third leaf from the top was collected from three randomly selected tomatoes from each cage. Frass and leaf samples were collected after approximately two weeks of herbivory. All samples were frozen in liquid nitrogen and stored at −80°C until processing. 16S rRNA gene sequencing and analysis was performed by Microbiome Insights (Vancouver, BC, Canada), using primers targeting the V4 region on an Illumina MiSeq, as per previous protocols^46^. Peptic nucleic acid (PNA) PCR clamps were included in all samples to limit the amplification of host chloroplast- and mitochondria-derived 16S rRNA genes.

Fastq files were quality-filtered and clustered into 97% similarity Operational Taxonomic Units (OTUs), using the mothur software package and its standard operational protocol^47^. Briefly, sequence pairs were concatenated and resulting sequences with lengths higher than 275 bases or ambiguous bases in them were removed. Sequences were then aligned against the Silva v132 master alignment, and trimmed to the region delimited by 13,862 and 23,444 positions. Chimeras were removed with uchime. Sequences were then classified with the Silva database using the RDP Naïve Bayesian classifier, and those classified as mitochondria, chloroplast, archaea, Eukaryota, or unknown were removed to account for non-specific amplification.

### Statistical analyses

Data analysis for terpenes, epicuticular waxes, and larval weight was performed on MVApp^40^. Briefly, data normality and variance were assessed using either the Bartlett’s or Levene’s tests. If these conditions were not satisfied, data was log-transformed to achieve normality. Further, we performed one-way and two-way ANOVA analyses to examine variation. Principal component analysis was performed in R using the *vegan* package^48^. OTUs were plotted in R to visualize the relative abundances of the bacteria at the phylum and genus level, and alpha- and beta-diversities^49^. We tested for difference among diets with PERMANOVA using *vegan*^48^. Negative binomial tests (DESEq2)^50^ were performed for differential OTU abundance analysis relative to plant host. Graphical representations were performed on R version 4.1.1^51^ with ggplot2^52^.

## Results

### Volatile terpene profiles varied with tomato genotype

**Figure 1:**
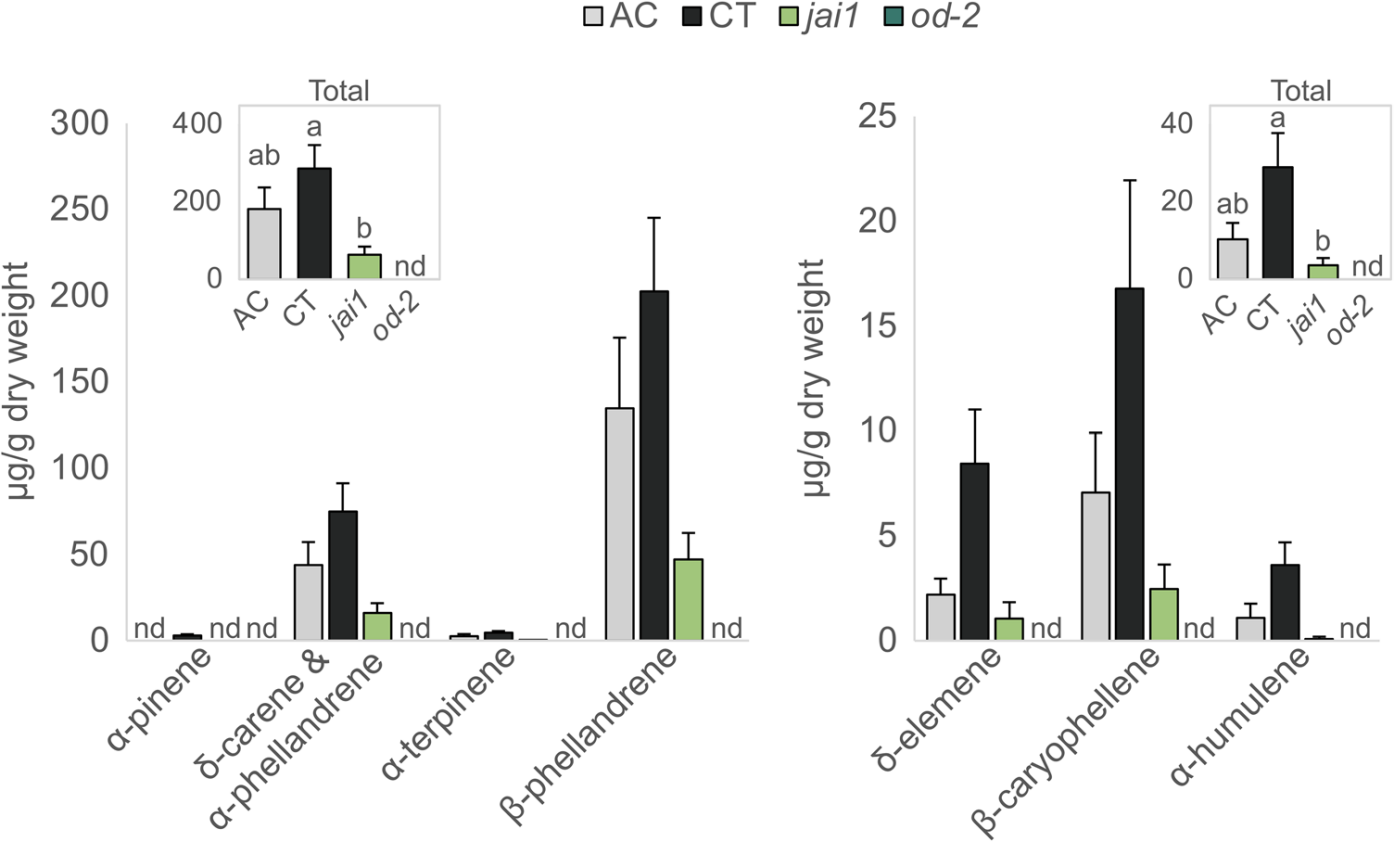
Basal volatile terpene levels in leaves of AC, CT, *jai1*, and *od-2*. Mono- (left panel) and sesqui- (right panel) terpenes were collected in hexane and run on GC-FID (method conditions led to coelution of δ-carene with α-phellandrene). Inset figures represent significant differences in the total average of monoterpenes (one-way ANOVA, *p* = 0.0336) and sesquiterpenes (one-way ANOVA, *p* = 0.0221) in each genotype. Three pairwise comparisons were done using a Tukey HSD test, with a Bonferroni adjustment for post-hoc comparisons. The *od-2* mutant was excluded from the comparison due to undetectable levels. The different letters indicate significant differences. Each data point represents the mean ± SE of four biological replicates. nd, not detected.

To assess the effect of leaf specialized metabolites on the insect microbiome, we first compared the leaf volatile terpene composition of the *jai1* and *od-2* mutants, their corresponding wildtype background (CT), and an additional tomato cultivar (AC). Under our experimental conditions, five monoterpenes (⍺-pinene, ∂-carene and ⍺-phellandrene, ⍺-terpinene, and β-phellandrene) and lower levels of three sesquiterpenes (∂-elemene, β-caryophyllene, and ⍺-humulene) were detected. Consistent with previous reports^34,35^, the volatile terpene levels of *jai1* and *od-2* were significantly affected, with a reduction of 75% observed in *jai1* and undetectable levels in *od-2* (Fig. 1). The two wildtype lines also differed in terpene accumulation, with AC accumulating approximately 40% less terpenes compared to CT. Despite their independent biosynthetic origins, mono- and sesquiterpene levels followed similar patterns. This reduction is expected given the trichome alterations observed in both mutants (Supplementary Fig. S1).

To assess potential changes in the leaf terpene accumulation upon insect feeding in the mutants, mono- and sesquiterpene levels were compared in systemic leaves over the course of 48 h of herbivory (Supplementary Fig. S2). Unexpectedly, the amount of terpenes did not increase in preformed leaves. Overall, the genotype and time had a significant impact on both monoterpene (two-way ANOVA, log_10_-transformed data, F_(3)time_ = 5.02, *p*_time_ = 0.0059; F_(2)genotype_ = 23.62, *p*_genotype_ = 5.85e-07) and sesquiterpene levels (two-way ANOVA, log_10_-transformed data, F_(3)time_ = 5.21, *p*_time_ = 0.0075; F_(2)genotype_ = 7.16, *p*_genotype_ = 0.0043). Upon insect feeding, *jai1* plants maintained their lower levels of accumulation compared to CT, and terpenes were still below detection in *od-2* plants despite having a functional jasmonate signaling pathway. These results indicate that, although there are dynamic changes in volatile terpenes with herbivory, the basal levels of preformed leaves are a good representation of the differences across genotypes.

### Additional surface compounds affected in tomato defence mutants

**Figure 2:**
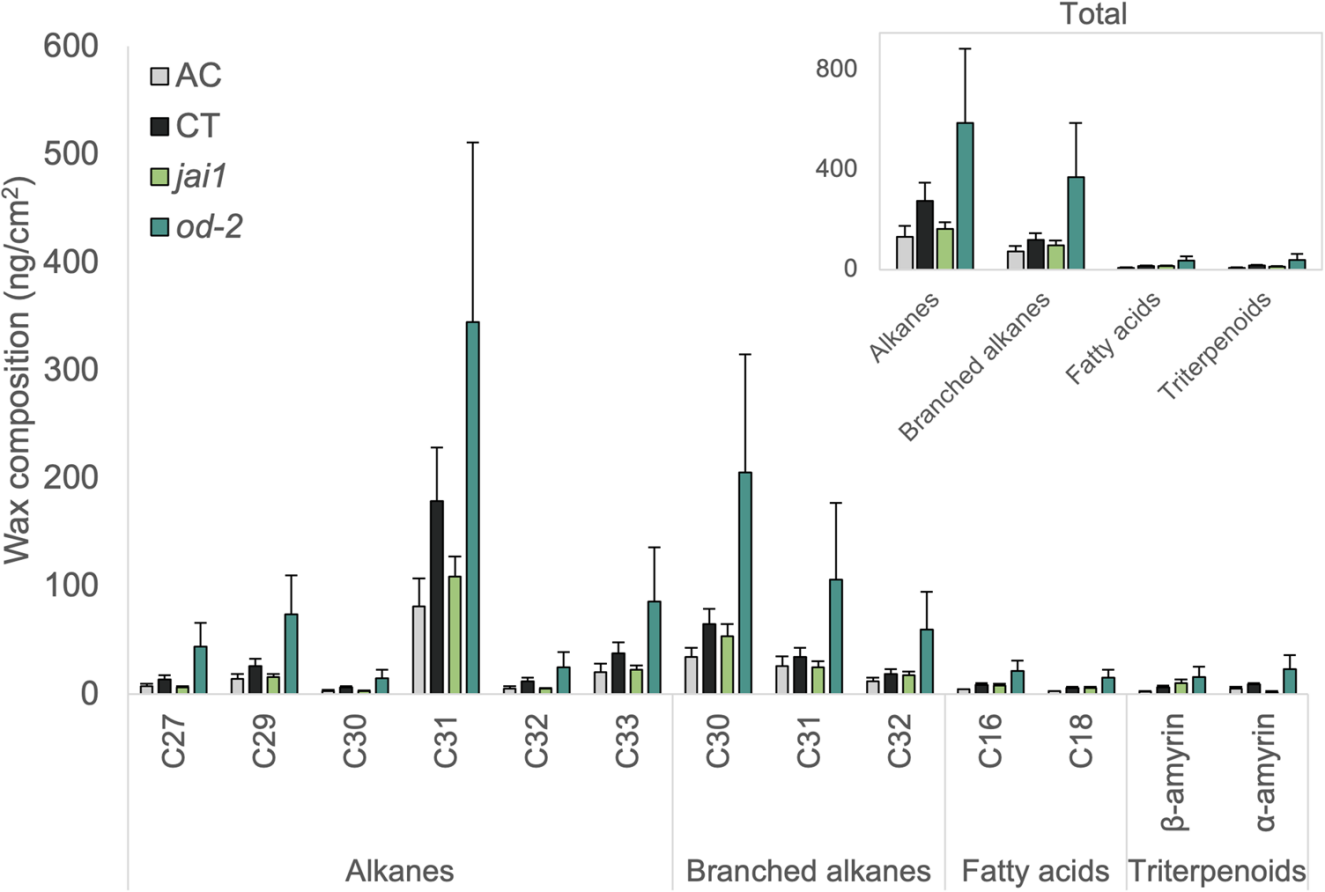
Basal foliar wax profiles of AC, CT, *jai1,* and *od-2*. Leaf wax compounds were grouped into four major classes: (1) alkanes, (2) branched alkanes, (3) fatty acids, and (4) triterpenoids. Inset graph indicates total wax amount for each chemical class. No significant differences were observed among genotypes in either of the classes (one-way ANOVA, *p*_alkanes_ = 0.276, *p*_branched alkanes_ = 0.332,* p*_fatty acids_ = 0.247, *p*_triterpenoids_ = 0.294). Each data point represents mean values ± SE (n_AC,CT_ = 3, n_*ja1,od-2*_ = 4).

To determine if the tomato genotypes also differ in their cuticular wax composition, larger non-polar lipids were collected. The leaf epicuticular waxes consisted of very-long-chain (VLC) alkanes (C27-C33) and methyl-branched alkanes (C30-C32), fatty acids (C16 and C18), and triterpenoids (⍺- and β- amyrin) (Fig. 2). Hentriacontane (C31) was the predominant component of foliar waxes in all genotypes, followed by 2-methyltriacontane (C30). Overall, the total load of alkanes and methyl-branched alkanes were lower in AC than CT (Fig. 2). The *jai1* mutation did not significantly affect cuticular wax accumulation, although lower levels of alkanes were present in *jai1* relative to CT. Interestingly, the *od-2* mutant seemed to accumulate higher amounts of alkanes and branched alkanes, as well as triterpenoids, although not significantly. The accumulation of triterpenoids in *od-2* contrasts with the severe defect in the accumulation of mono- and sesquiterpenes.

Upon herbivory, similar trends were observed for different compound classes regardless of whether they were derived from very-long-chain fatty acids (alkanes, branched alkanes and fatty acids) or from isoprenyl diphosphates (triterpenoids) (Supplementary Fig. S2). No significant differences were observed over time when compounds were combined into classes, except for free fatty acids. Overall, time had a significant impact on the VLC fatty acids levels for both wildtypes (two-way ANOVA, log_10_-transformed data, F_(3)_ = 6.53, *p* = 0.0029). Whereas genotype played a more significant role in the VLC fatty acids trends observed in the mutants relative to their background cultivar (two-way ANOVA, log_10_-transformed data, F_(__2)_ = 3.08, *p* = 0.017). The *od-2* mutant generally maintained higher levels of all compound classes relative to CT (Supplementary Fig. S3), whereas the *jai1* showed consistently, but not significantly, lower levels than CT.

### Insect performance is influenced by plant host

**Figure 3:**
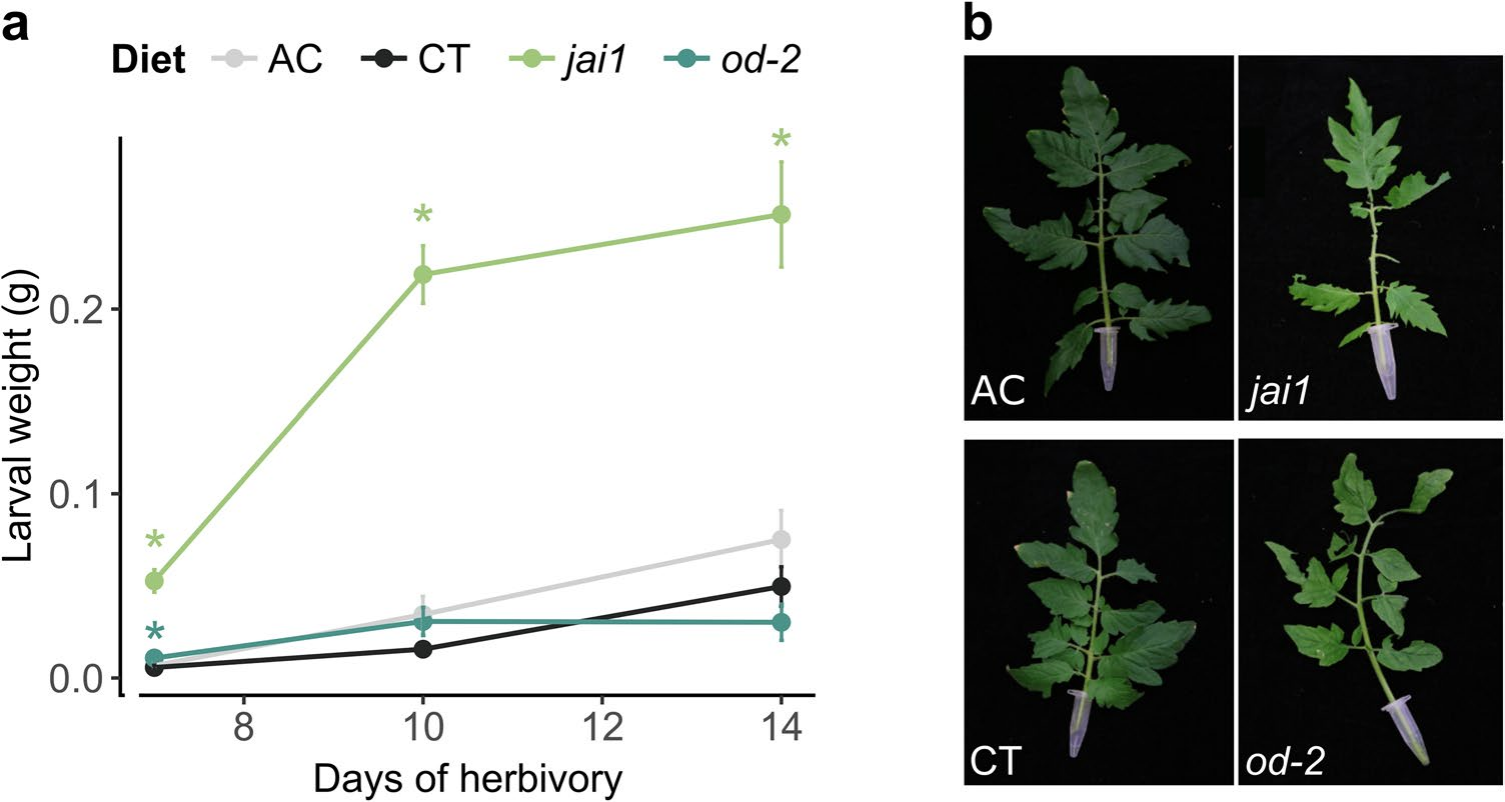
Performance of cabbage loopers on four tomato genotypes. (a) Insect performance timeline relative to diet source at 7, 10, and 14 days of herbivory. Larval weight was significantly impacted by the diet source (two-way ANOVA, F = 115.795, *p* < 2.2e^−16^) and time (two-way ANOVA, F = 46.887, *p* < 2.2e^−16^). Six pairwise comparisons of the log_10_-transformed data were done using a Tukey HSD test, with a Bonferroni adjustment for post-hoc comparisons. The stars represent significant differences between the defence mutants relative to the background cultivar, CT. No significance was observed between caterpillars fed on AC and CT. (**b**) Representative damage after 48 h of feeding by two last-instar caterpillars. Caterpillars were grown on wheat germ diet until the fourth instar before placing on a detached leaf from each genotype.

To test if the mutations in *jai1* and *od-2* translate to differences in insect performance under our experimental conditions, neonate larvae were allowed to feed ad libitum and weighed. The interaction between plant host and feeding time had a significant impact on the insect performance (two-way ANOVA, F_(6, 313)_ = 22.095, *p* < 2.2e−16) (Fig. 3a). Despite the differences in volatile terpene and wax content, insects reared on both wildtype cultivars gained similar weight. Seven days after feeding, caterpillars reared on *jai1* and *od-2* were significantly heavier than their wildtype counterparts. However, at later time points, the caterpillars reared on *od-2* performed similarly to those reared on the wildtype cultivars, whereas the ones on *jai1* consistently gained more weight.

Within the cage setting, insects reached the last instar at different time points depending on their host plant, complicating comparisons of tissue damage. Hence fourth instar caterpillars were placed on a detached leaf from each genotype to compare tissue consumption. After 48 h, damage is evident on all leaves, yet the *jai1* showed more defoliation relative to the other genotypes (Fig. 3b). This indicates that the increased weight sustained by the mutant plants is accompanied by more leaf damage by last instar caterpillars.

### Fatty acid composition altered during insect digestion

**Figure 4:**
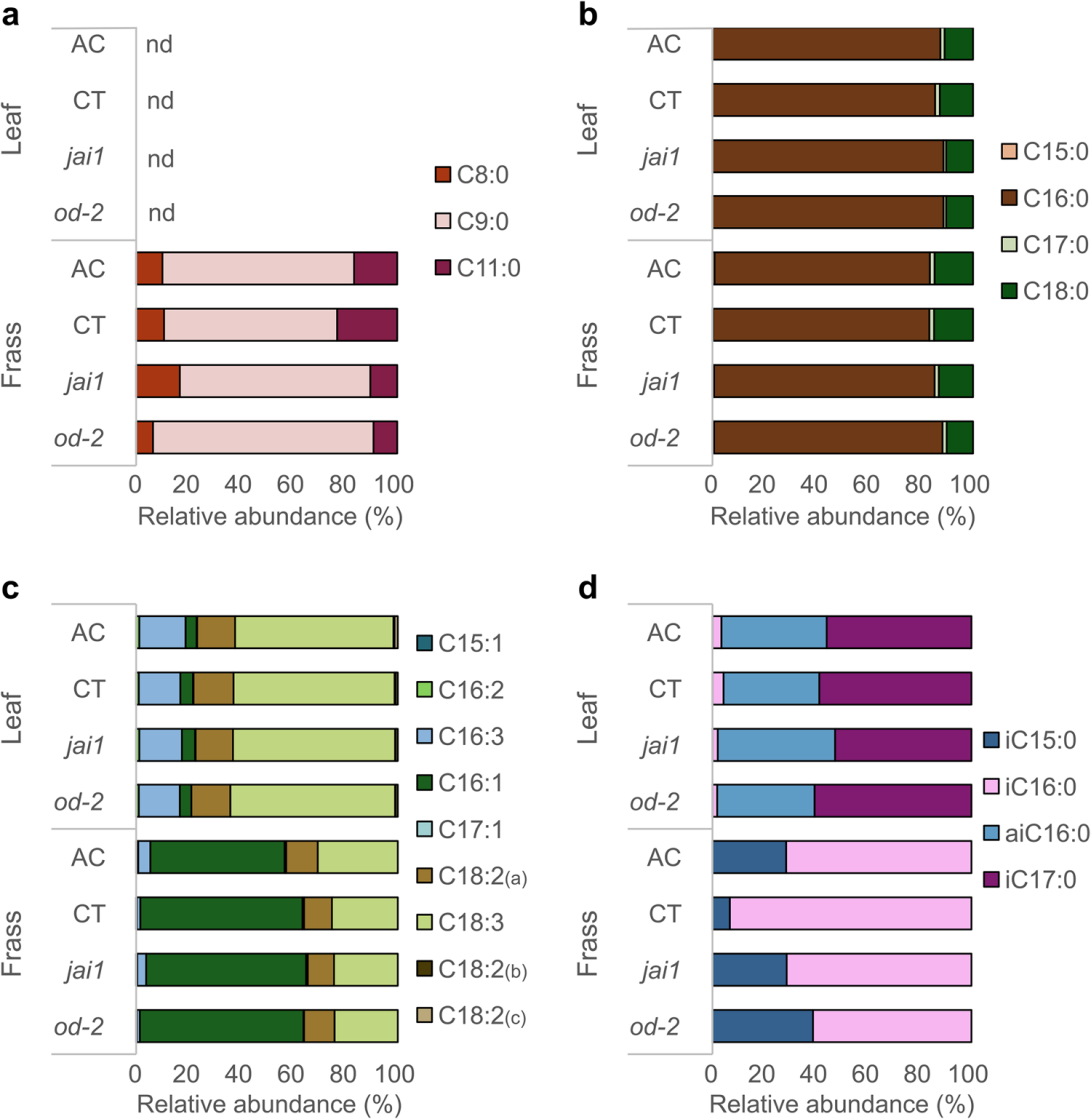
Variation in relative abundance of fatty acids in leaf and frass samples. Fatty acids measured as fatty acid methyl esters were grouped into four classes: (**a**) medium chain saturated, (**b**) long chain saturated, (**c**) long chain unsaturated, and (**d**) branched long chain saturated. Each bar represents the relative mean of four biological replicates. nd, Not detected.

In this study, we focused on measuring lipidic specialized metabolites (terpenes and waxes); however, we did not know if primary metabolites were also affected in the mutants. To tackle this issue, we used fatty acids, an important component of the insect diet, to survey the changes that occur during digestion by comparing the fatty acid composition of the food (leaves) and end-product of digestion (frass). The leaf fatty acid composition consisted of three saturated, eight unsaturated, and three branched long-chain fatty acids (Fig. 4). Unlike the differences in specialized metabolites, no significant differences were found across plant genotypes, indicating that the mutations in plant defences present in *jai1* and *od-2* are not likely affecting lipid primary metabolism.

**Figure 5:**
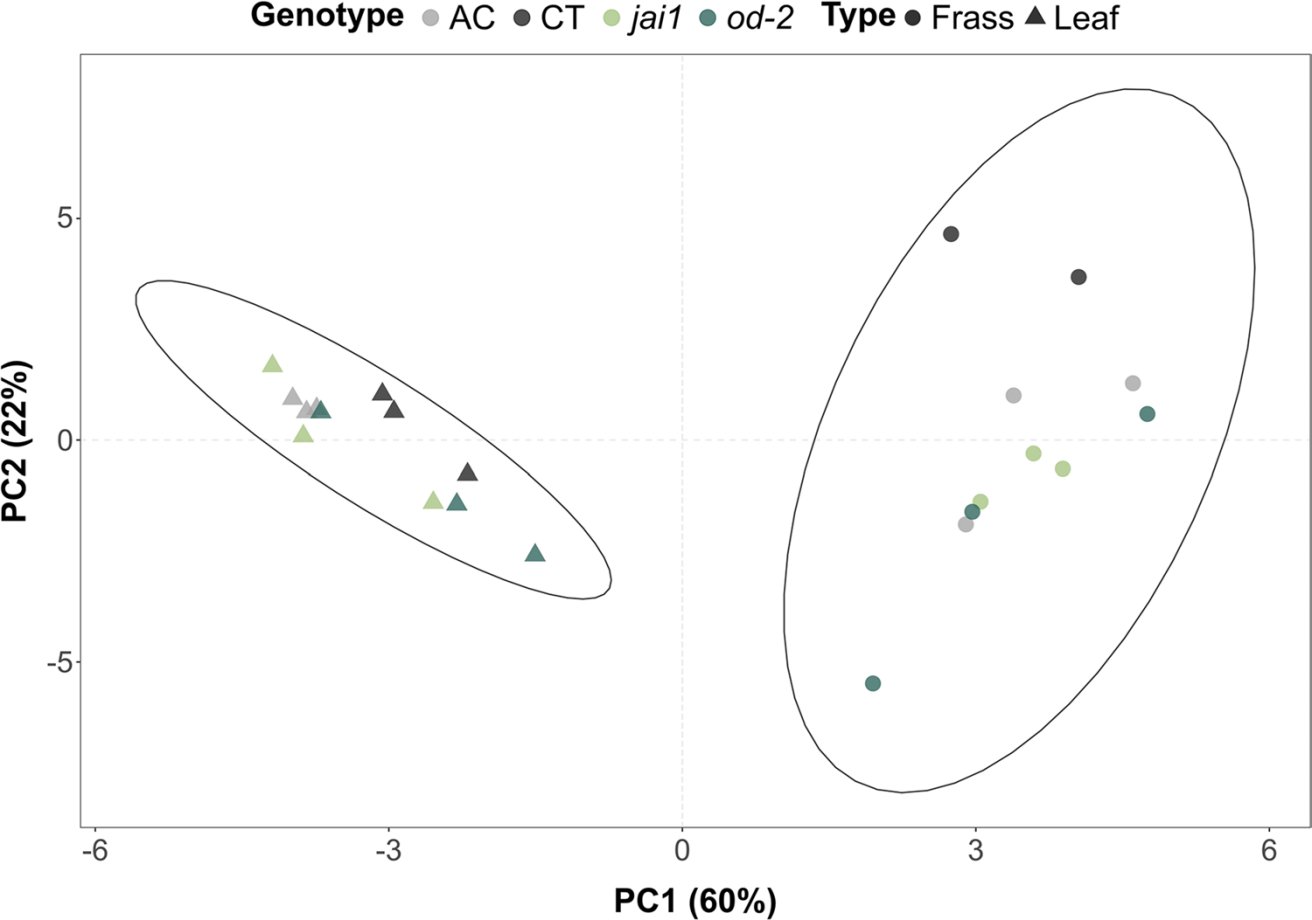
Principal component analysis (PCA) of fatty acids from leaf and frass samples. Two-dimensional PCA score plots reveal separation driven by sample type. Ellipses represent the 95% confidence interval. Based on the 1.5 IQR rule, one outlier was identified and removed in the frass samples (n_leaf_ = 12 and n_frass_ = 11).

Frass contains a mixture of fatty acids produced by the plant, the insect, and also by microbes. Most compounds were shared between leaf and frass samples, except for three saturated medium-chain fatty acids, pentadecanoic acid, and pentadecenoic acid that were unique to frass samples. Meanwhile, two isomers of octadecadienoic acid were identified exclusively in the leaf. Palmitic acid (C16) and stearic acid (C18) were the predominant compounds in leaves and frass but did not differ in quantity (Fig. 4b). Overall, there seems to be a tendency towards shorter fatty acids in the frass as expected from catabolic processes. However, the leaves had a higher relative abundance of saturated long chain fatty acids than frass (Supplementary Fig. S4). Differences in the relative abundance of unsaturated and branched long chain fatty acids were also observed between frass and leaves (Fig. 4c, d; Supplementary Fig. S4a). Overall, leaf samples cluster closer to each other and separately from frass samples (Fig. 5). This indicates that although food retention time for Lepidopterans is short, there are large changes in fatty acid composition as plant material is digested.

Interestingly, insects fed on CT had higher quantity of fatty acids in their frass (Supplementary Fig. S4b). These insects were exposed to an active jasmonate signalling pathway and to the largest amount of terpenes and waxes in their diet. The detected amounts of medium-chain saturated fatty acids were similar in frass samples from caterpillars fed on AC and CT (Fig. 4a). However, insects fed on *jai1* and *od-2* had a 56% and 61% reduction, respectively, in undecanoic acid (C11) levels relative to the insects fed on the background cultivar. In contrast, pentadecanoic (C15) and 14-methyl-pentadecanoic acid (iC15:0) acid relative abundance levels were elevated 50% in *jai1* and *od-2* relative to the amounts detected in CT (Fig. 4b, d). This indicates that the genotype of the host plant, not only affects larval weight, but also disturbs insect digestive metabolism of lipids.

### Insect bacterial communities did not mirror leaf bacterial communities

The short food retention time and relatively simple insect digestive tract of Lepidopterans has been used to question the functionality of the insect gut bacterial microbiome^53^. If this is true, we hypothesized that frass bacterial communities would be a close reflection of the bacterial communities present on the leaf. To test this, leaf and frass bacterial communities were compared to determine the extent of the contribution of the leaf microbiome to the frass. 16S rRNA genes were amplified from frass and the insect’s corresponding diet source, generating a total of 823,749 reads. Leaf samples represent only approximately 37% of the total reads indicating possible under sampling; however, strong ecological drivers in bacterial populations can still be identified^54^.

**Figure 6:**
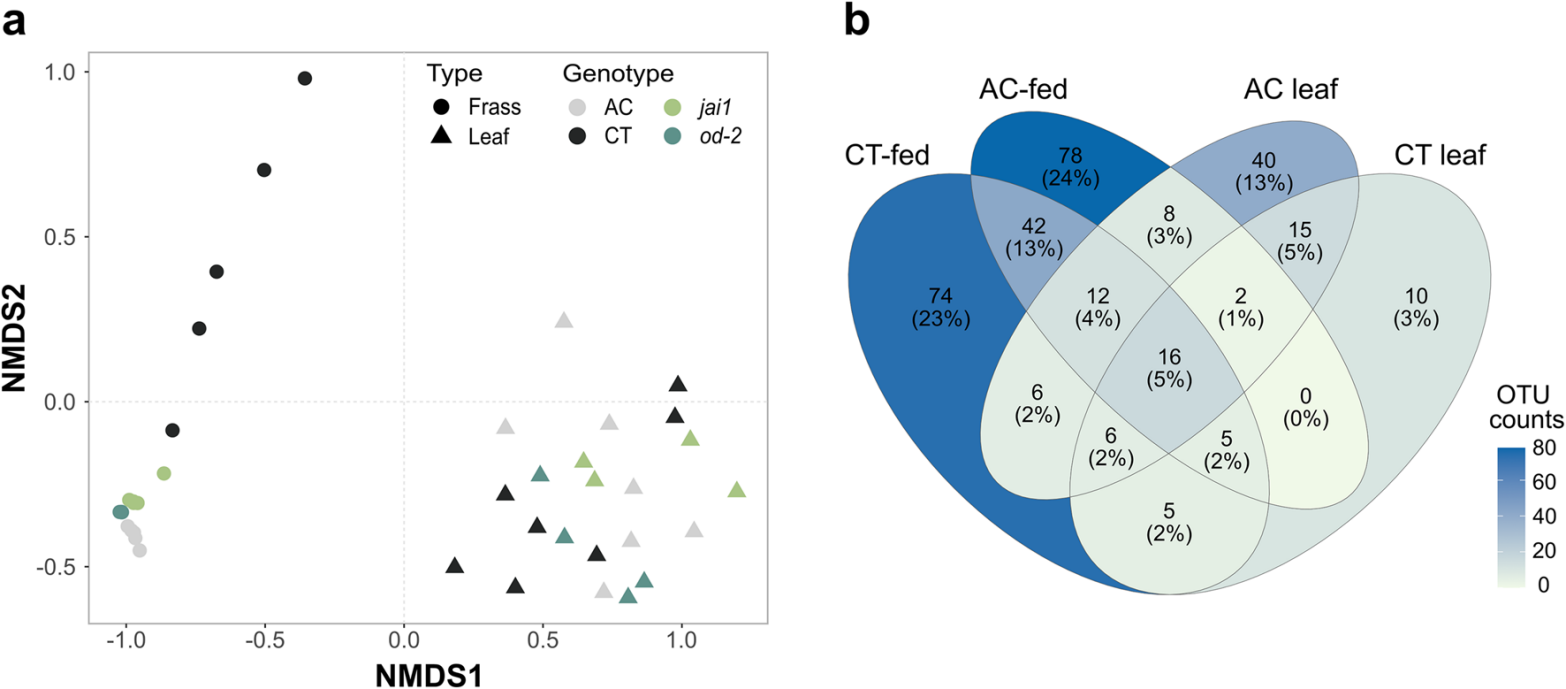
Comparison of bacterial community composition. (**a**) A Bray–Curtis dissimilarity index was used to compare frass and leaf samples. Non-metric multidimensional scaling (NMDS) analysis separates samples based on sample type and genotype. The separation between the communities was significantly impacted by both genotype and sample type (PERMANOVA, n = 4–7, *p* = 2e−04). (**b**) Shared and unique OTUs found in AC and CT leaves and frass from insects reared on those genotypes.

Non-metric multidimensional scaling (NMDS) was used to visualize similarity between samples. The analysis showed that leaf and frass bacterial communities are clearly separated (Fig. 6a). A Permutation Multivariate Analysis of Variance (PERMANOVA) indicated that both sample type (frass or leaf) and genotype (AC, CT, *jai1* and *od-2*) had a significant impact on segregation of bacterial communities. To investigate the diversity within the communities, alpha diversity was estimated by comparing the Chao1, ACE, Shannon, and Simpson indexes (Supplementary Table S1). Leaf bacterial communities had many diverse groups, with few dominating bacterial genus (Supplementary Fig. S5). In contrast, frass samples had reduced bacterial diversity as their communities were dominated by members of Enterobactericeae, *Enterobacterales*, Yersinaceae, and *Chrysoeobacterium*.

The frass and leaf bacterial communities of the two wildtype cultivars (AC and CT) were further compared for common taxa. Only 5% of the OTUs were shared between the four samples (Fig. 6b). Within the 5%, we found bacterial members of Enterobacteriaceae OTU0001, *Pseudomonas* OTU0002, and *Acinetobacter* OTU0003. Similar pairwise comparisons between the insect-associated bacteria and their corresponding diet source for each of the four genotypes indicated that on average only 13.75 ± 1.97% OTUs are shared (Supplementary Table S2. Altogether, this indicates that although the insects are feeding entirely on tomato, their gut bacterial microbiome is not an exact reflection of the bacterial communities present on the leaf.

### Larval gut bacterial microbiomes varied across tomato genotypes

**Figure 7:**
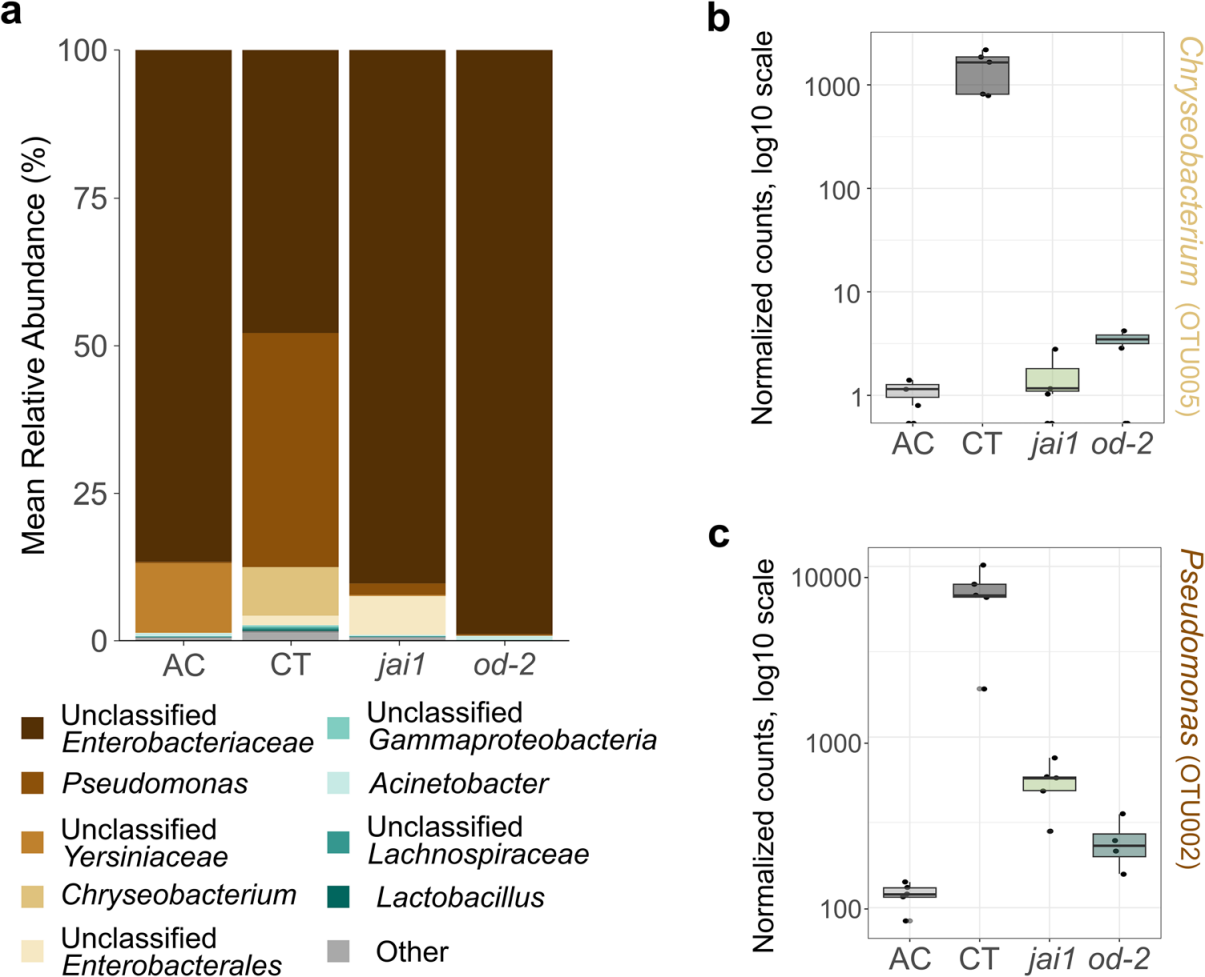
Frass bacterial community composition as function of host plant genotype. (a) The bar plot displays the average bacterial genus abundance of five replicates. Depicted bacterial genera accounted for at least 2% of the total, otherwise they were categorized as “Other”. (**b, c**) Relative abundance of the two most predominant bacteria genera was compared across samples relative to their diet source using DESeq (*p* < 0.05; log_10_-transformed data).

In addition to differences in plant specialized metabolite composition and insect growth among genotypes, we also observed differences in the fatty acid profiles and bacterial communities of plants and insect guts. Next, we tested if gut bacterial communities change relative to genotype insects were reared on. The insect-associated OTUs were classified into three main phyla: Bacteroidota, Firmicutes, and Proteobacteria. Proteobacteria was predominant in all insects regardless of their diet source, accounting for over 90% of the abundance across all frass samples. However, at the genus level, differences in bacterial abundance between the two wildtype cultivars as well as between the mutants and their background cultivar were observed (Fig. 7a). Bacterial groups belonging to unclassified genera in the Enterobacteriaceae family predominated across the samples. The unclassified Enterobacteriaceae group made up 48% of the bacterial abundance in insects reared on CT, and over 85% in insects reared on AC (87%), *jai1* (90%), and *od-2* (99%). Significant differences were observed across frass samples in unclassified *Chryseobacterium*, *Pseudomonas*, Yersiniaceae, *Acinetobacter*, and unclassified *Enterobacterales* (*p* < 0.001 for each) (Supplementary Table S3). Based on OTU counts, *Chryseobacterium* OTU005 and *Pseudomonas* OTU002 were more abundant in CT relative to the mutants and the other wildtype cultivar (Fig. 7b, c).

**Figure 8:**
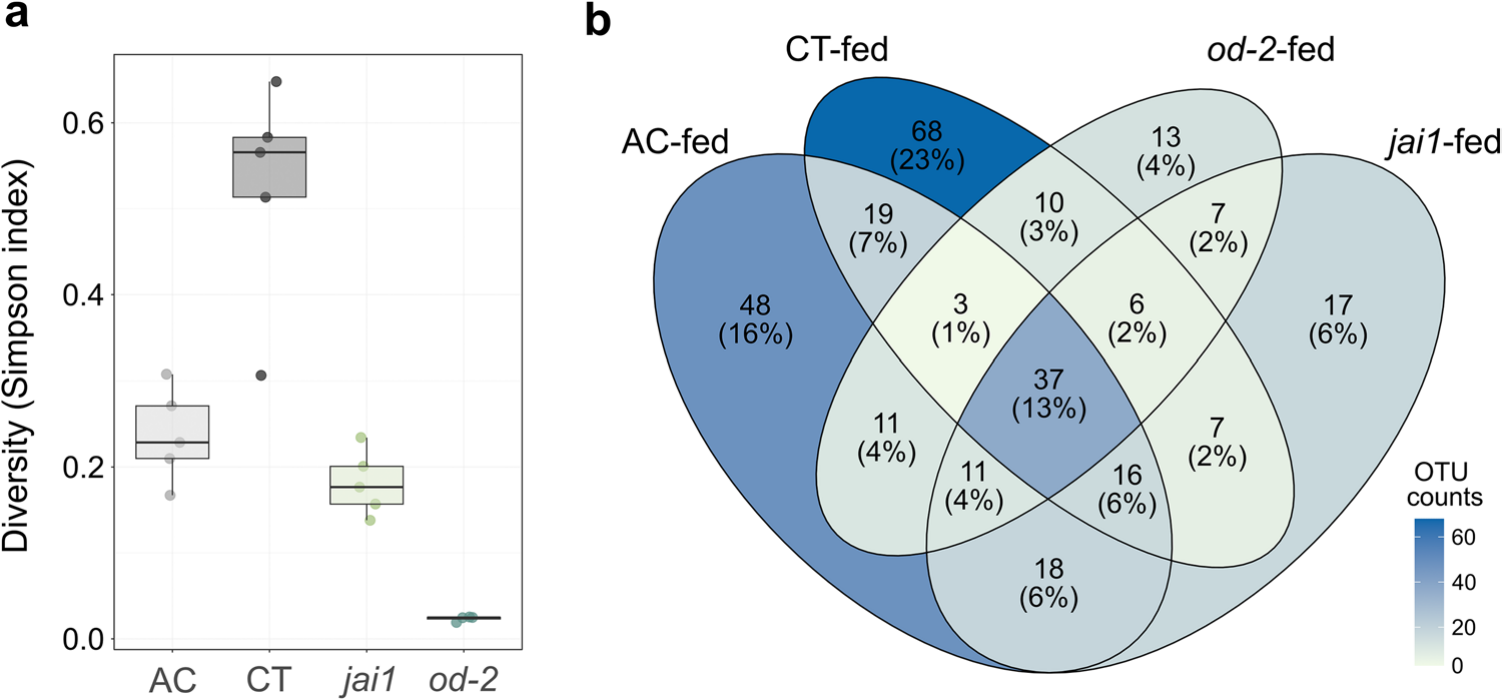
Diversity of frass bacterial communities relative to the diet of the caterpillars. (**a**) The Simpson index shows significant differences between insects reared on different genotypes (ANOVA, *p*=0.0001). (**b**) Shared and unique OTUs from insect frass relative to the diet source.

We further tested for differences in alpha and beta diversity in the frass bacterial communities across plant genotypes. The Simpson diversity index was highest in the samples of insects fed CT relative to all the others (Fig. 8a). The insects reared on *od-2* had a Simpson index of 0.023 ± 0.002, suggesting that their community structure was dominated by few bacterial groups (Supplementary Table S4). Based on Bray–Curtis dissimilarity indexes, the genotype of the plant on which the insects fed on had a significant impact on driving the segregation between the frass bacterial communities (Fig. 6a). Insects fed on CT had scattered clustering compared to the ones fed on either the mutants or AC. However, clear clustering patterns relative to diet source were observed, supporting that the plant genotype is a key modulator of the insect gut bacterial microbiome composition. Interestingly, insects fed on genotypes not impacted in defences had more unique OTUs relative to the mutants (23% unique OTUs in CT and 16% in AC compared to 6% in *jai1* and 4% in *od-2*) (Fig. 8b). Bacterial members of *Cyanobacteriia* OTU0020, *Streptococcus* OTU0045, and Lachnospiraceae OTU0054, along 16 other OTUs, were shared among insects fed on the two wildtypes, but not found in the mutant-fed insects. Moreover, only 2% of the OTUs were shared among insects fed on CT and the two defence-deficient mutants. Overall, this suggests that the presence of plant defences is associated with increased diversity of taxa found in the frass.

## Discussion

### Tomato defences affect insect growth

The damage and increased weight on the mutants were proportional to the defect in plant defences in *jai1* and *od-2*. JAs signaling coordinates the biosynthesis of many specialized metabolites, one class being terpenes^23,34,55^. In contrast, despite the severe reduction in terpenes in *od-2,* other defensive metabolites and proteins still accumulate. After seven days of feeding, insects reared on *od-2* gained more weight than those on wildtype, but the difference in growth was not maintained at later time points. This suggests that metabolites that are impaired in *od-2* are particularly important in defending plants at early stages of infestation and/or when larvae have recently hatched. This is consistent with the defects of *od-2* on several constitutive surface-related structures and chemicals^34^. Interestingly, although the insects reared on the *od-2* mutant caught up in growth with the ones on WT after 10 days, we could still detect differences in the bacterial communities present in larvae from the later instars. This suggests that the effects of plant defences on bacterial communities are not transient and emphasize the need to include additional measurements in plant–insect interactions studies, since insect weight only provides a partial picture. In contrast, insects reared on the *jai1* mutant showed increased weight across 14 days, expected from the larger defects observed upon disruption of JA signaling. Structures and specialized metabolites present in the epidermis are likely to be more relevant in early stages of plant–insect interactions, before induction of jasmonate defences.

The *jai1* and *od-2* mutants have been previously reported to have defects in the accumulation of defensive metabolites and trichomes, without major effects on growth or development^34,35^. As expected, the defence-deficient mutants, had lower terpene accumulation than their background cultivar (CT). In *od-2* and *jai1,* the levels of mono- and sesquiterpenes are reduced; however, similar levels of triterpenes were detected in their epicuticular waxes relative to the background CT. This could indicate that the biosynthesis of triterpenes is under independent control in epicuticular waxes, and/or the supply of isoprenoid substrates is not the limiting factor for the emission of volatile terpenes in either *od-2* or *jai1*. In addition to the major defects in trichomes, *od-2* has irregularly-shaped and raised epidermal pavement cells^34^. The data presented here indicates that this phenotype is accompanied by an increase in cuticular waxes, particularly linear and branched alkanes. It is worth noting that to be emitted into the atmosphere, terpenes produced in trichomes and epidermal cells must cross the cuticle; hence alterations in the cuticle can have downstream effects on emission of terpenes^56^. It is possible that the odorless phenotype is a consequence of a larger developmental defect in epidermal cells, including trichomes and cuticular waxes coating epidermal cells. As the first line of defence against the environment, disentangling the multiple strategies present in epidermal cells and their impact on insect herbivory requires further exploration.

Because the phenotypes in the mutants could go beyond the specialized metabolites screened here and previously reported ^34,35^, we were interested in testing if the total fatty acid profile was affected as a proxy of differences in primary metabolism. Dietary fatty acids are essential in insect metabolism, playing roles in fat body, oocyte (eggs), and hormone synthesis^57,58^. Overall, there were no significant differences in the leaf fatty acid composition, unlike the differences observed in specialized metabolites, suggesting that the nutritional value was similar among cultivars and genotypes. As plant lipids are digested, they are hydrolyzed by lipases into free fatty acids, absorbed in the midgut, and further oxidized in the mitochondria for energy production^57^. Interestingly, even though leaf samples had similar fatty acid composition, frass samples showed some differences depending on the genotype the insects were fed on. Compared to leaf samples, frass samples had an approximate 10% and 50% reduction in long-chain saturated and branched fatty acids, respectively. These observations suggest that insect digestion of fatty acids can be modulated by plant defences.

### Frass and leaves share limited bacterial taxa

Leaves are not homogenous surfaces, they are covered by a waxy cuticle, trichomes and a diversity of defensive compounds contained therein. These conditions can shape the structure of the phyllo- and endosphere bacterial communities^59,60^. Similarly, the insect gut provides a challenging habitat for microbial colonization, especially considering the alkaline conditions and that the midgut’s peritrophic matrix changes with every molt^61^. Overall, little is known about the mechanisms involved in bacterial colonization in the Lepidopteran gut. As insects consume leaves, their guts are exposed to the plant microbiome, which made us hypothesize that this would be the major source of bacterial communities^53^. However, our findings suggest that only a small percentage of bacteria are shared between plant and caterpillar gut bacterial microbiomes. These observations are consistent with previous reports in other Lepidopterans fed on tomato and *Brassica* spp.^62,63^, and hint at additional mechanisms for colonization. We used insects that were provided as surface-sterilized eggs, hence maternal transmission of bacteria is expected to be minimal. Moreover, seeds were also surface-sterilized before being planted in autoclaved growing media and grown in the same greenhouse environment, providing a homogenous environment for bacterial communities to colonize; rather than an environment with well-established resident bacteria from the egg, seed and soil. It is worth noting that, despite the range of conditions under which similar studies have been conducted, there is a core set of taxa that are consistently found associated with insect guts, including Enterobacteriaceae, *Pseudomonas*, *Acinetobacter,* and *Enterococcus*^31^. Even under these homogenizing conditions, insect gut bacterial communities were not a mere reflection of the leaf microbiome, supporting that physicochemical conditions of the gut are a crucial component in shaping the bacterial communities. How those conditions are changed by plant structural and chemical defences, and whether the effect is direct or indirect by affecting insect physiology requires further investigation^44,61,64^.

The cabbage looper gut bacterial community was predominantly composed of bacteria belonging to the Enterobacteriaceae family. This is consistent with previous findings in cabbage loopers reared on wheat germ diet, *Arabidopsis thaliana, S. lycopersicum,* and *Brassica oleracea*^28,44,62^*,* suggesting that Enterobacteriaceae spp. may be a constituent group*.* Some Enterobacteriaceae spp. have been shown to respond to terpene exposure^28^. In our studies, we noticed that the more terpenes a plant contains, the lower relative abundance of Enterobacteriaceae spp. Moreover, several Enterobacteriaceae isolates from *Spodoptera exigua* (Lepidoptera) have been shown to modulate JA-mediated defences in tomato by downregulating polyphenol oxidase and trypsin protease inhibitor activity, hence benefitting insect digestion^65^. In silkworm larvae (Lepidoptera), *Enterobacter* spp. (Enterobacteriaceae) contribute to the prevention of pathogen colonization^11^. In addition, Enterobacteriaceae OTUs were also found in leaves from AC and CT. In the case of shared OTUS, it would be interesting to follow up the colonization. The shared OTUs could be introduced from the plant to the insect by feeding, or introduced from the insect to the plant by regurgitation. This has been previously observed in *Enterobacter* isolates from *Helicoverpa zea* (Lepidoptera), where inoculation by regurgitation promotes tomato plant growth and yield without compromising anti-herbivore defenses^66^. In the particular case of the large Enterobacteriaceae spp. group, it would be important to improve the taxonomic resolution within this taxon before speculating on the function to the insect host. Amplicon sequencing is a valuable tool, yet it can underestimate the full diversity present in the samples^67^.

### Insects feeding on plants with more defences have increased gut bacterial diversity

Despite having predominant groups, the bacterial communities in the cabbage looper gut were sensitive to the changes in plant defences presented in the mutants. For example, CT plants contained more trichomes and specialized metabolites, and as expected, insects reared on CT had poor growth. But this was also accompanied by an increased number of unique OTUs identified in their frass and a larger amount of total fatty acids. This could indicate that insect digestion has been negatively affected by the defences present in the plant, whether directly by impairing insect metabolism, or by shaping the gut bacterial microbiome remains to be determined. Additionally, we observed a pattern between the foliar terpene quantity and frass bacterial alpha diversity. Overall, alpha diversity was lower in insects that fed on plants with reduced terpenes. Although they varied in their bacterial community census, insects reared on AC and *jai1* had similar diversity indexes. Interestingly, the terpene and wax loads of AC and *jai1* were also similar. With minimal exposure to terpenes, the bacterial community of insects fed on *od-2* was almost entirely composed of *Enterobacteriaceae*.

Additionally, our results show that *Chryseobacterium* spp. and *Pseudomonas* spp. were significantly more abundant in caterpillars with poor performance that had been reared on tomatoes with intact defences. *Chryseobacterium* spp. are known to be overrepresented in terpene exposed caterpillars and induce higher mortality in *Protaetia brevitarsis seulensis* (Coleoptera) due to their pathogenic nature^68,69^. Bacteria of the genus *Pseudomonas* inhabit cabbage looper midguts and have been linked to weaker immune systems in other insects^44,70,71^. However, the presence of *Pseudomonas* spp. may also indicate symbiotic relationships between insects and bacteria. For instance, the Colorado potato beetle (Coleoptera) uses *Pseudomonas* symbionts to supress tomato defences^10^. Our results suggest that plant defences may promote the colonization of the gut with a more diverse microbiome, including bacteria with known negative effects on insect growth. It is worth noting that mutations in the biosynthesis of specialized metabolites can often result in pleiotropic effects in other pathways^72^. Therefore, it is not clear if the affected bacterial communities are a direct consequence of structural and chemical defences affected in the mutants, or other non-described physiological defects. Future studies are needed to establish the relation between terpenes and certain OTUs; for example, by overexpression or knock-out of a terpene synthase. While we observed an interaction between plant genotype and insect microbiome, we cannot yet decouple the effects that the plant genotype has on bacterial communities.

In summary, our findings demonstrate that plant defences can have an influence on the insect gut bacterial microbiome. This study brings a thorough and unique perspective into the plant–insect-microbiome system by incorporating plant-specialized metabolite profiling, genotypic variation, frass lipid profiling and microbial community analyses. Insect gut bacterial communities are an integral component of plant–insect interactions, and a better mechanistic understanding of their roles is needed to utilize them in pest control practices.

### Supplementary Information

Supplementary Information 1.

Supplementary Information 2.

## Data Availability

The 16S rRNA gene dataset generated during this study is deposited at NCBI SRA repository, under accession number PRJNA1006157 https://dataview.ncbi.nlm.nih.gov/object/PRJNA1006157?reviewer=ucffsjlcbebg142qc66bg47vbl.
