# Supplementary material for: Tomato defences modulate not only insect performance but also their gut microbial composition
Source: Sci Rep. 2023 Oct 24;13:18139. doi: 10.1038/s41598-023-44938-2 (PMC10598054; doi:10.1038/s41598-023-44938-2)
Supplement: Supplementary file 1 — Supplementary Information 1. [file 41598_2023_44938_MOESM1_ESM.docx]

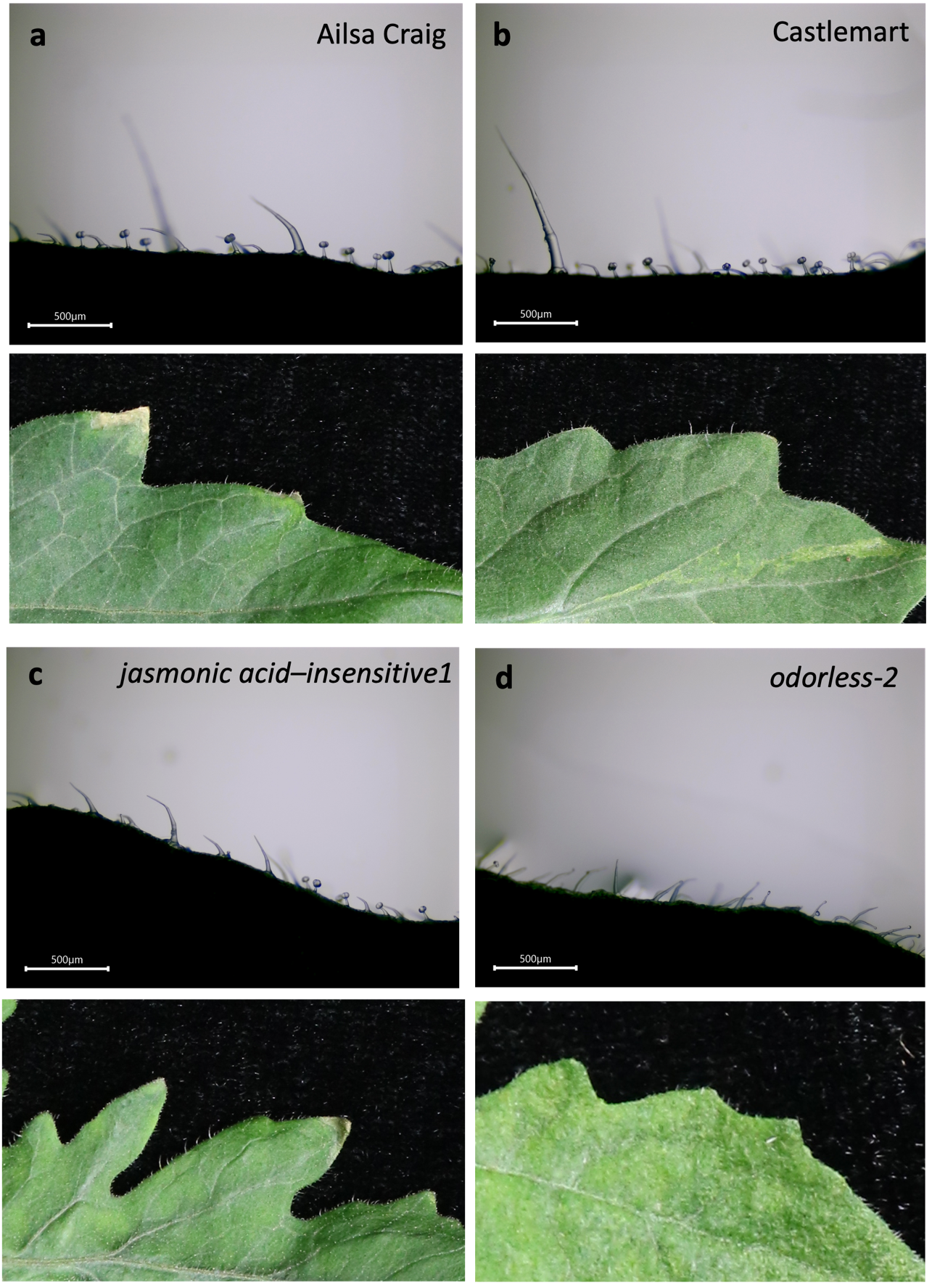


**Supplementary Figure 1.** Representative leaf images of the four genotypes, including light microscopy images of the trichomes. The fourth youngest leaf of two-week old plants was photographed, and one leaflet from each leaf was observed under a light microscope under 4X. bar = 500µm.

**Supplementary Figure 2.** Total leaf volatile terpenes in systemic tissue upon herbivory by cabbage loopers. The mono- and sesquiterpene levels of the two wildtype cultivars (AC and CT; **a, c**) and the defence-deficient mutants and their background cultivars (CT, *jai1*, *od-2*; **b, d**) were compared. The *od-2* had non-detectable amounts of terpenes across the 48 h sampled. Each data point represents the mean ± SE of four biological replicates. Time points reflect hours of exposure to herbivory (1 = 0 h; 2 = 8 h; 3 = 12 h; 4 = 48h).

**Supplementary Figure 3.** Leaf wax compound classes upon herbivory by cabbage loopers. The compound class levels of the two wildtype cultivars (AC and CT; **a-d**) and the defence-deficient mutants and their background cultivar (CT, *jai1*, *od-2*; **e-h**) were compared. Genotype played a significant role in the differences observed between the mutants and CT (two-way ANOVA, log_10_-transformed data, F_(2)alkanes_=8.29, *p_alkanes_* << 0.001; F_(2)branched alkanes_=10.32, *p_branched alkanes_* << 0.001; F_(2)fatty acids_=4.61, *p_fatty acids_*=0.017; F_(2)triterpenoids_=6.64, *p_triterpenoids_*=0.0039). The x-axis represents categorical variables). Each data point represents the mean ± SE of four biological replicates. Based on the 1.5 IQR rule, two outliers were identified and removed in the *od-2* samples (n=2). Time points reflect hours of exposure to herbivory (1 = 0 h; 2 = 8 h; 3 = 12 h; 4 = 48h).

**Supplementary Figure 4.** Total fatty acid content in leaf and frass samples represented as (**a**) relative abundance by compound classes as represented in **Figure 4**, and (**b**) total amount. No saturated medium chain fatty acids were detected in leaf samples. The total fatty acid amounts did not vary among genotypes (one-way ANOVA, p_Leaf_=0.622, p_Frass_=0.071). Each bar represents the mean of three biological replicates. MCFA = Medium Chain Fatty Acids; LCFA = Long Chain Fatty Acids.


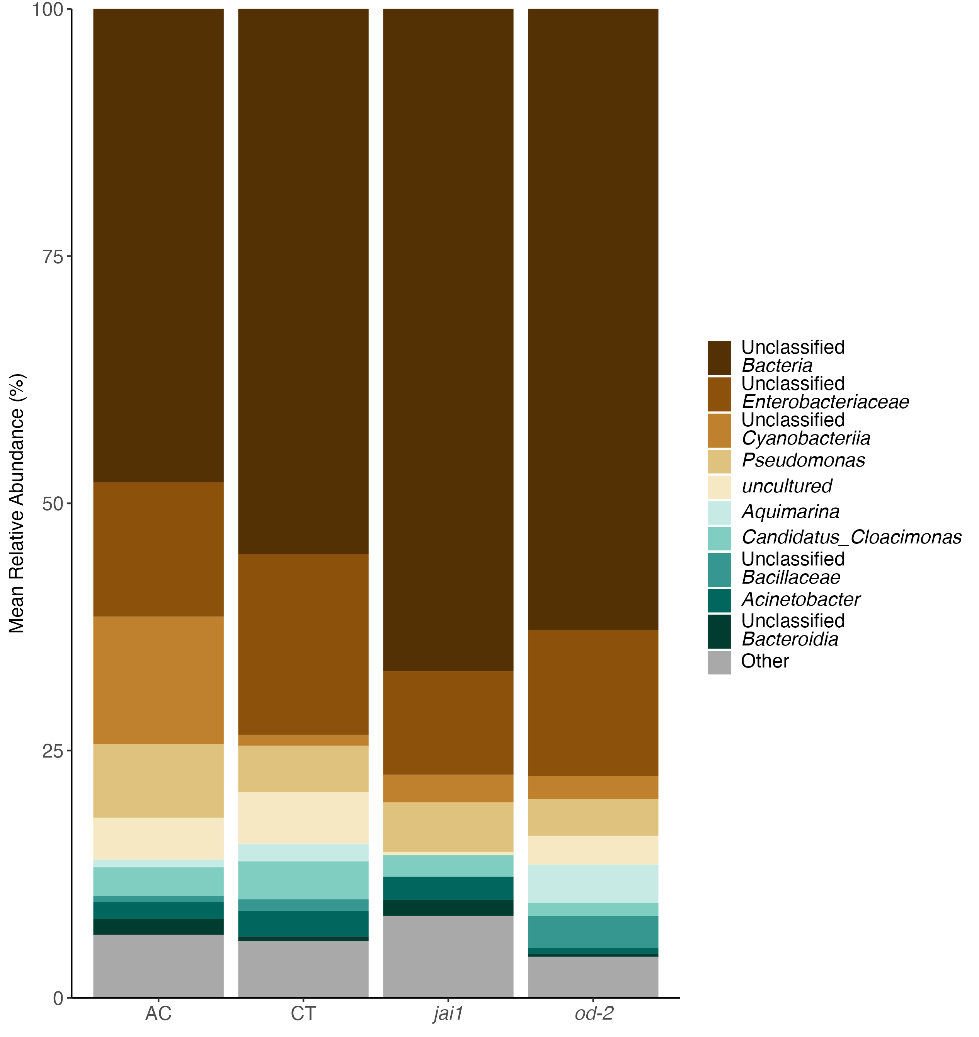


**Supplementary Figure 5.** The mean relative abundance of bacteria genera in leaf samples, classified by plant genotype. The depicted bacterial genera made up at least 3% of the total relative abundance, otherwise they were categorized as “Other”.
